# Supplementary material for: Improved High-Quality Genome Assembly and Annotation of Pineapple (Ananas comosus) Cultivar MD2 Revealed Extensive Haplotype Diversity and Diversified FRS/FRF Gene Family
Source: Genes (Basel). 2021 Dec 24;13(1):52. doi: 10.3390/genes13010052 (PMC8774480; doi:10.3390/genes13010052)
Supplement: Supplementary file 1 [file genes-13-00052-s001.zip › Supplementary Figures Document genes.pdf]

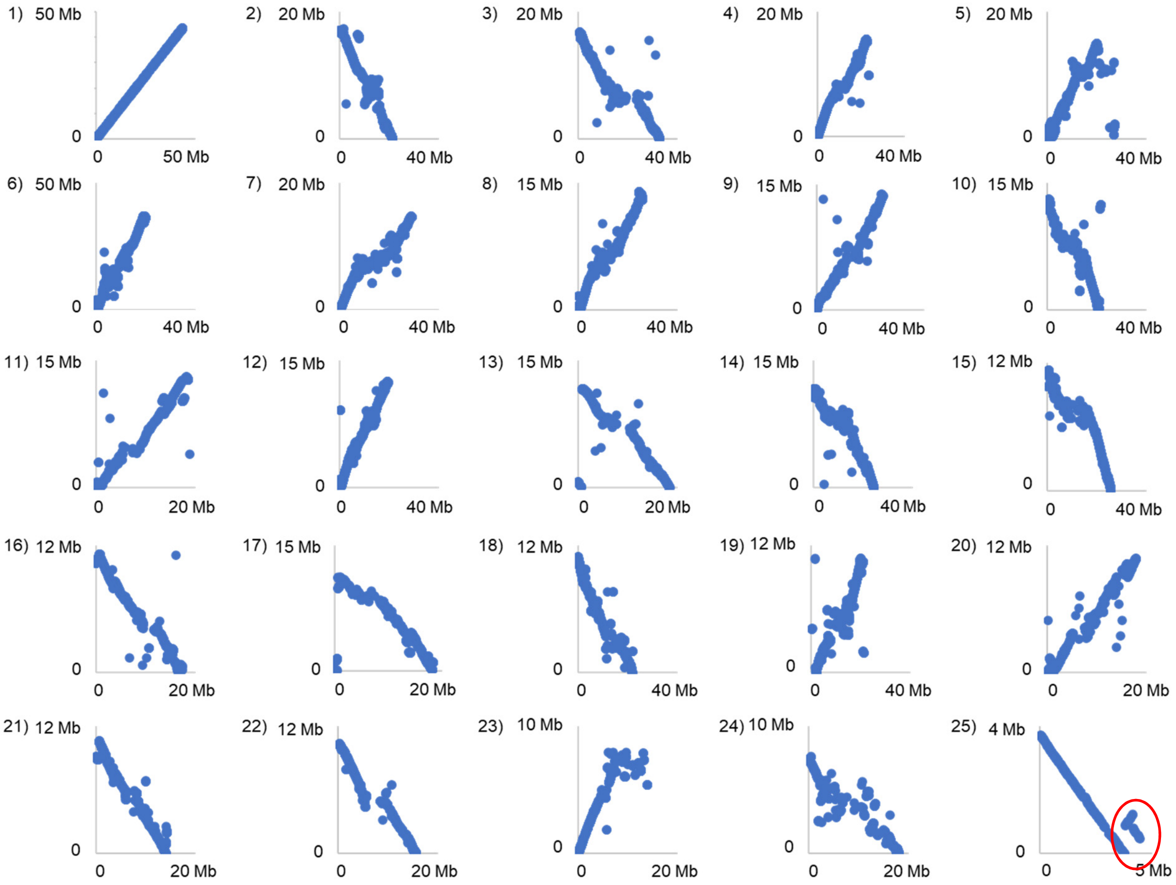

Figure S1. Alignment of the SSR marker sequences representing the F153 linkage map against the MD2 v2 chromosomes. For each panel, the y-axis corresponds to the genomic locations of the SSR markers in the F153 genome and the x-axis corresponds to the genomic locations of the SSR markers in the MD2 v2 genome. Chromosome number is indicated in top left of each panel. Non-collinear regions (eg. red circle) between the F153 SSR linkage map and the MD2 v2 assembly represent cases where higher confidence during the manual correction of the MD2 v2 chromosomes was given to the Hi-C interaction data over the SSR map.

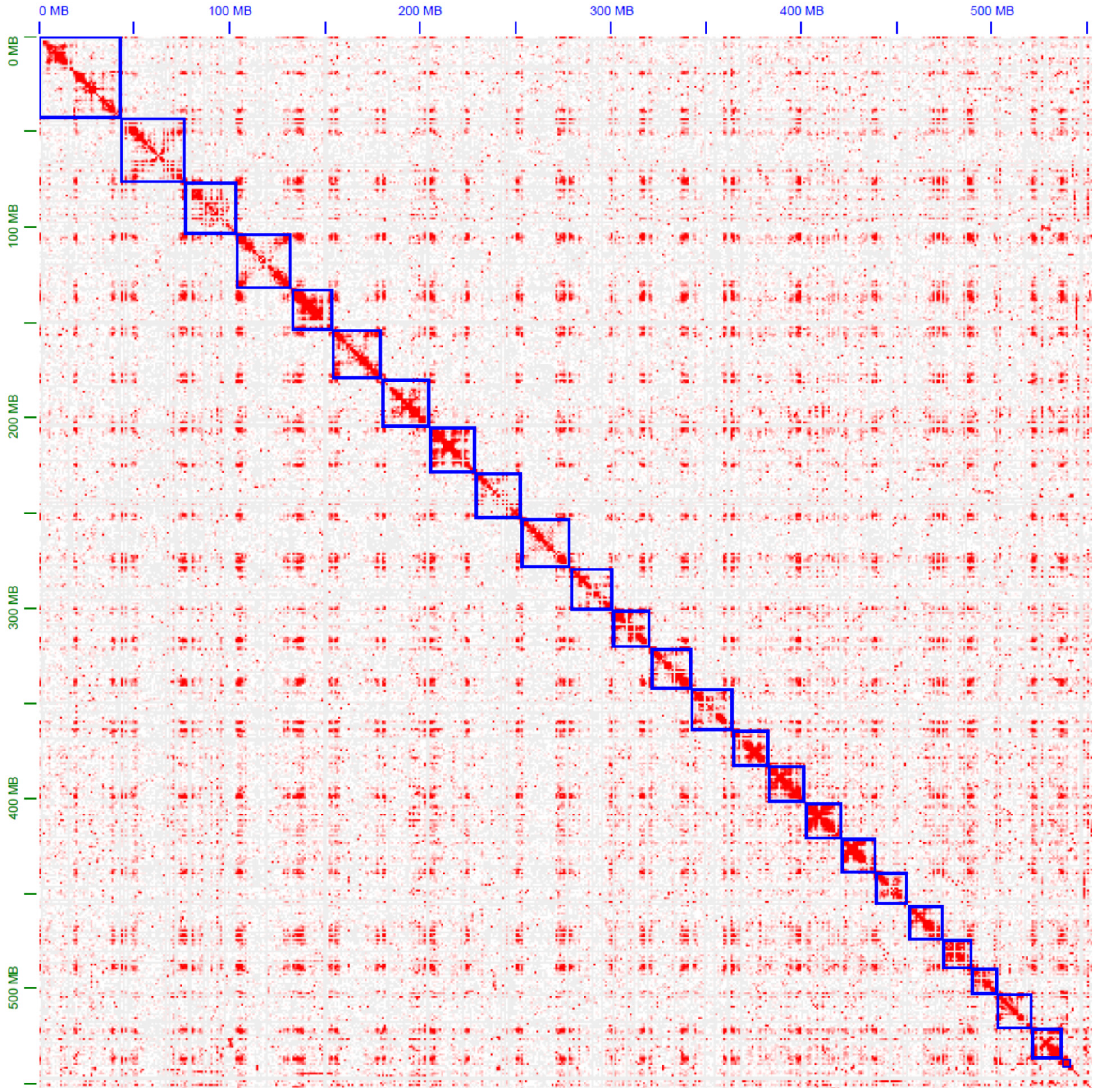

Figure S2. MD2 v2 assembly Hi-C heatmap showing a uniform distribution of genomic interactions along the diagonal (indicated by red color on the diagonal). The red coloring demonstrates the proximity of the assembled sequences and the quality of the assembly. Blue boxes indicate whole chromosomes (25 total).

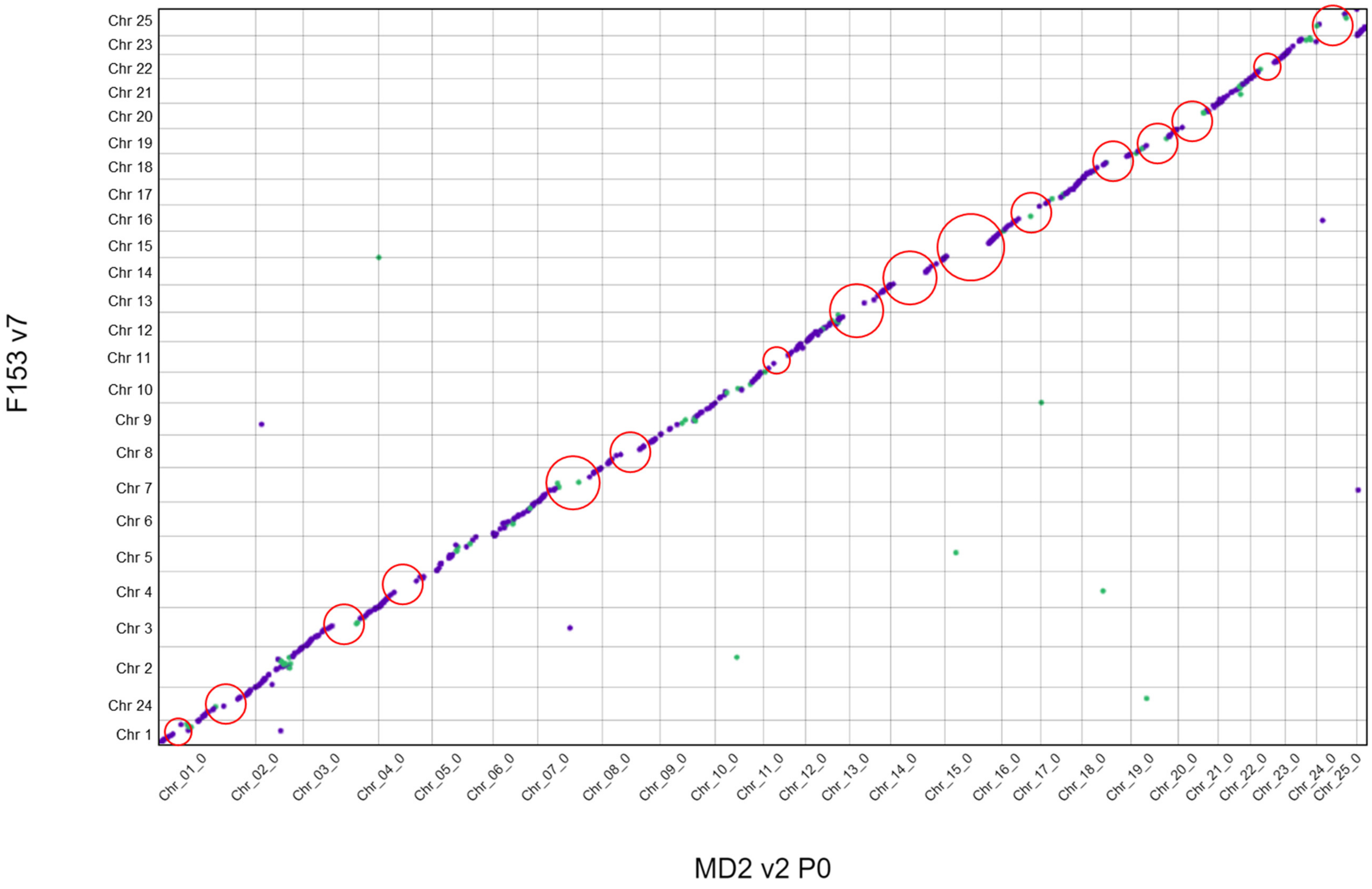

Figure S3. Nucmer alignment of the MD2 v2 P0 and F153 v7 genome assemblies. Large gaps are circled in red to highlight improvements in the MD2 v2 assembly.

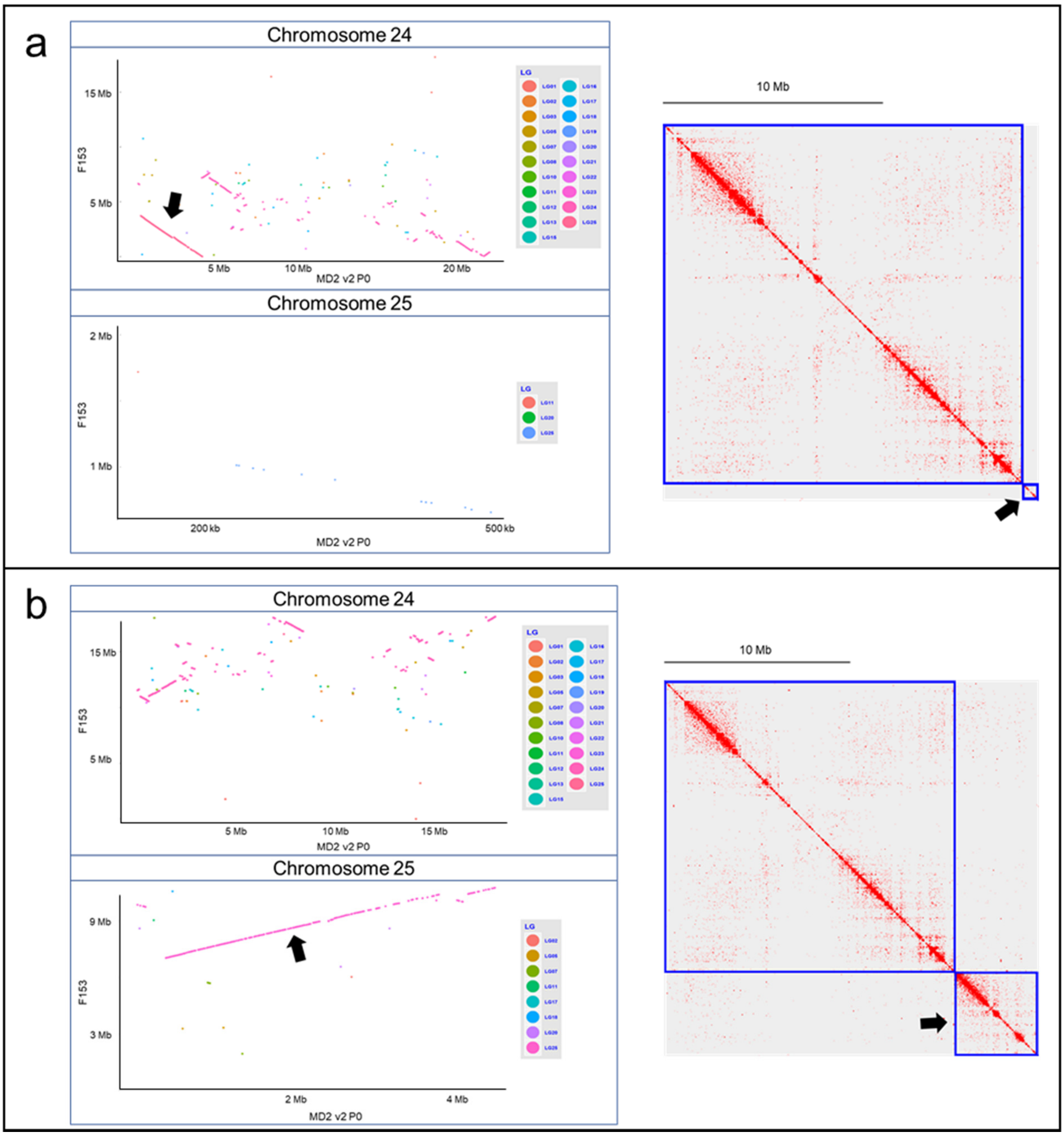

Figure S4. SSR linkage map (left) and Hi-C data (right) before (a) and after (b) correction of MD2 v2 chromosome 25. (a) A fragment of chromosome 25 was initially assembled into chromosome 24. (b) Based on the SSR linkage map, and Hi-C data it was manually corrected and placed into chromosome 25. Black arrows in the SSR linkage map plot indicate genomic regions belonging to chromosome 25. Black arrows in the Hi-C data plot indicate chromosome 25 before (a) and after (b) correction of the chimeric region.

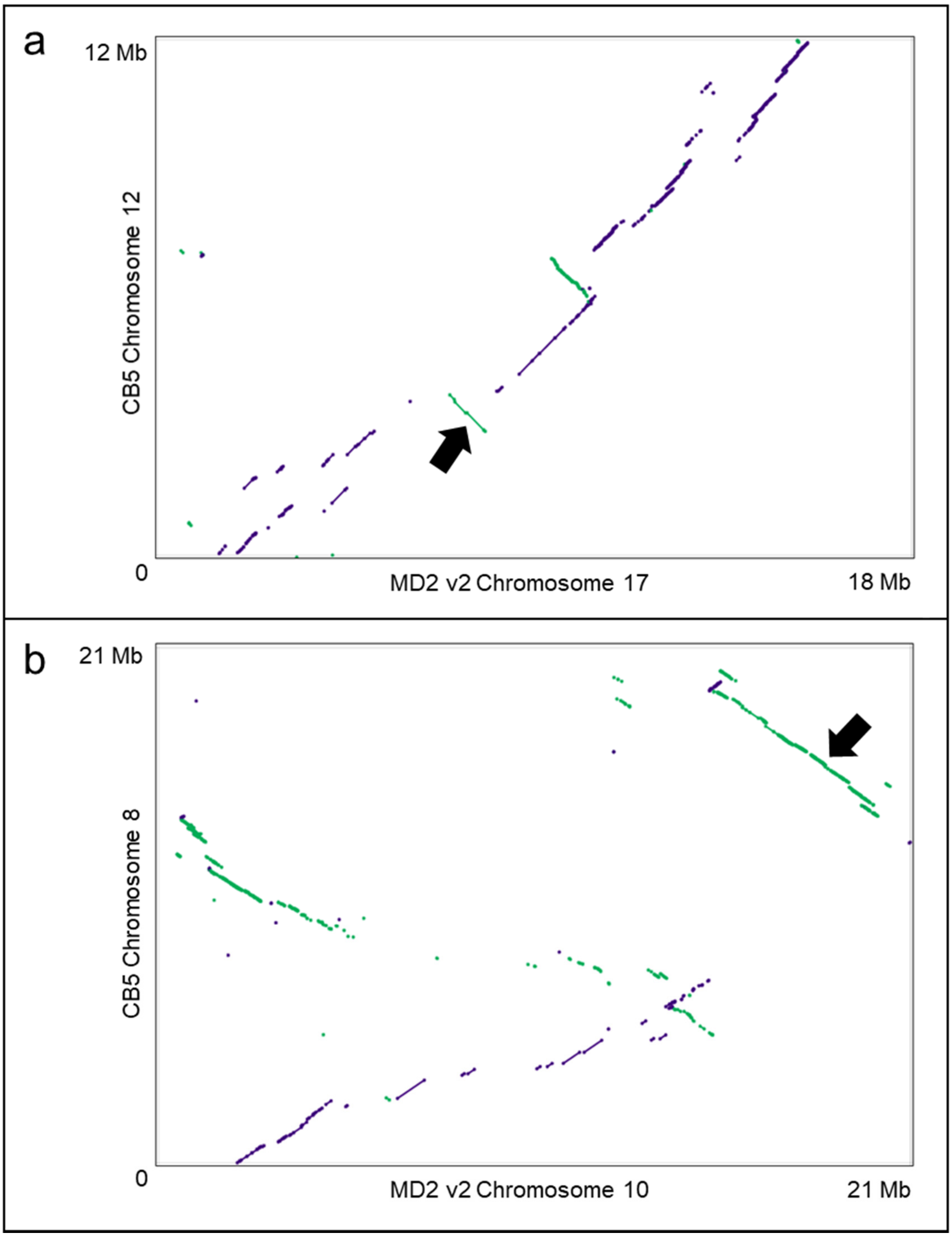

Figure S5. Small (a) and large (b) inversions in CB5 chromosome 12 and chromosome 8, respectively.

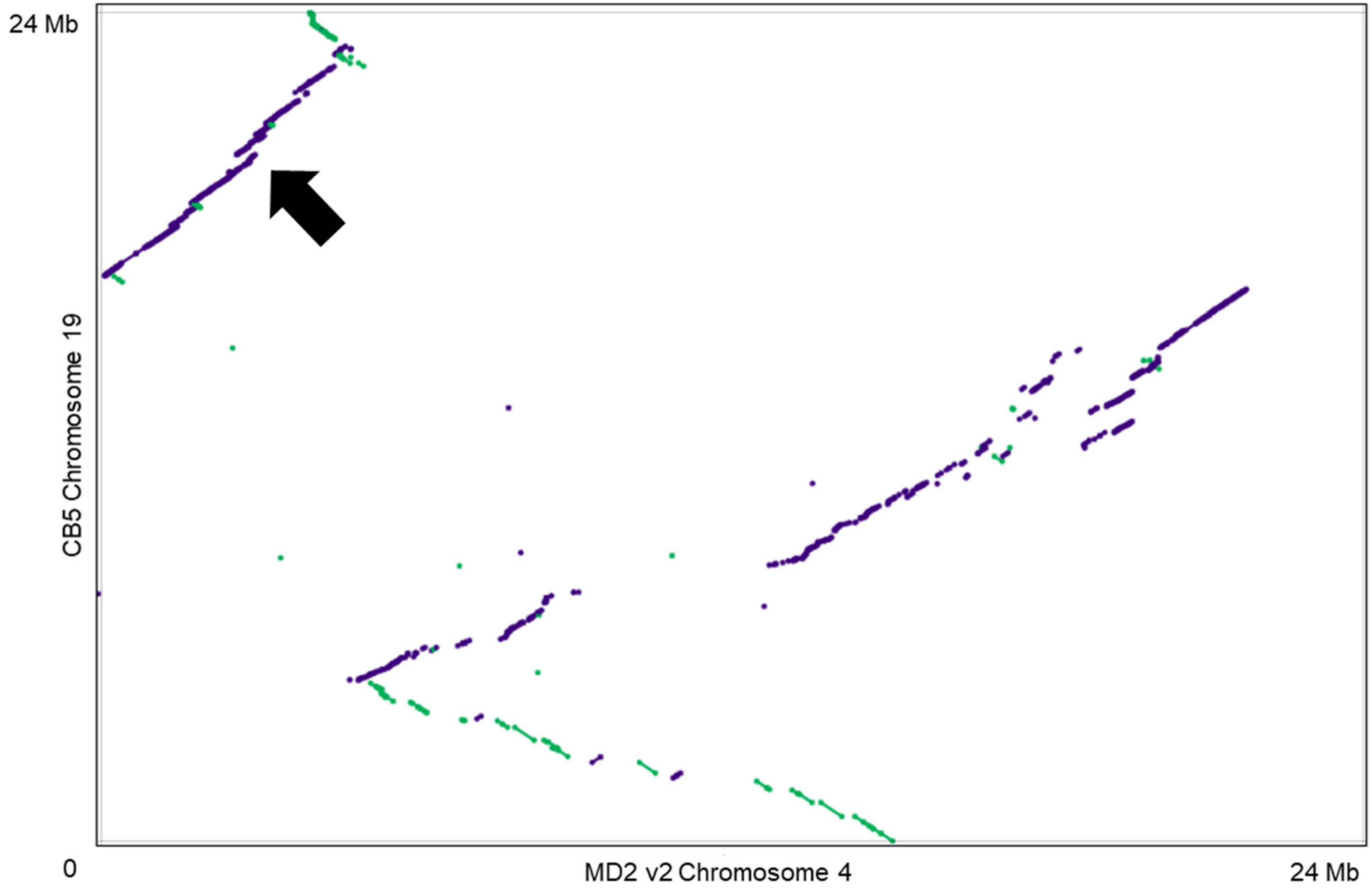

Figure S6. Example of intrachromosomal translocation in CB5 chromosome 19. The black arrow points to a genomic region located at the beginning of MD2 v2 chromosome 4, but is located at the end of CB5 chromosome 19.

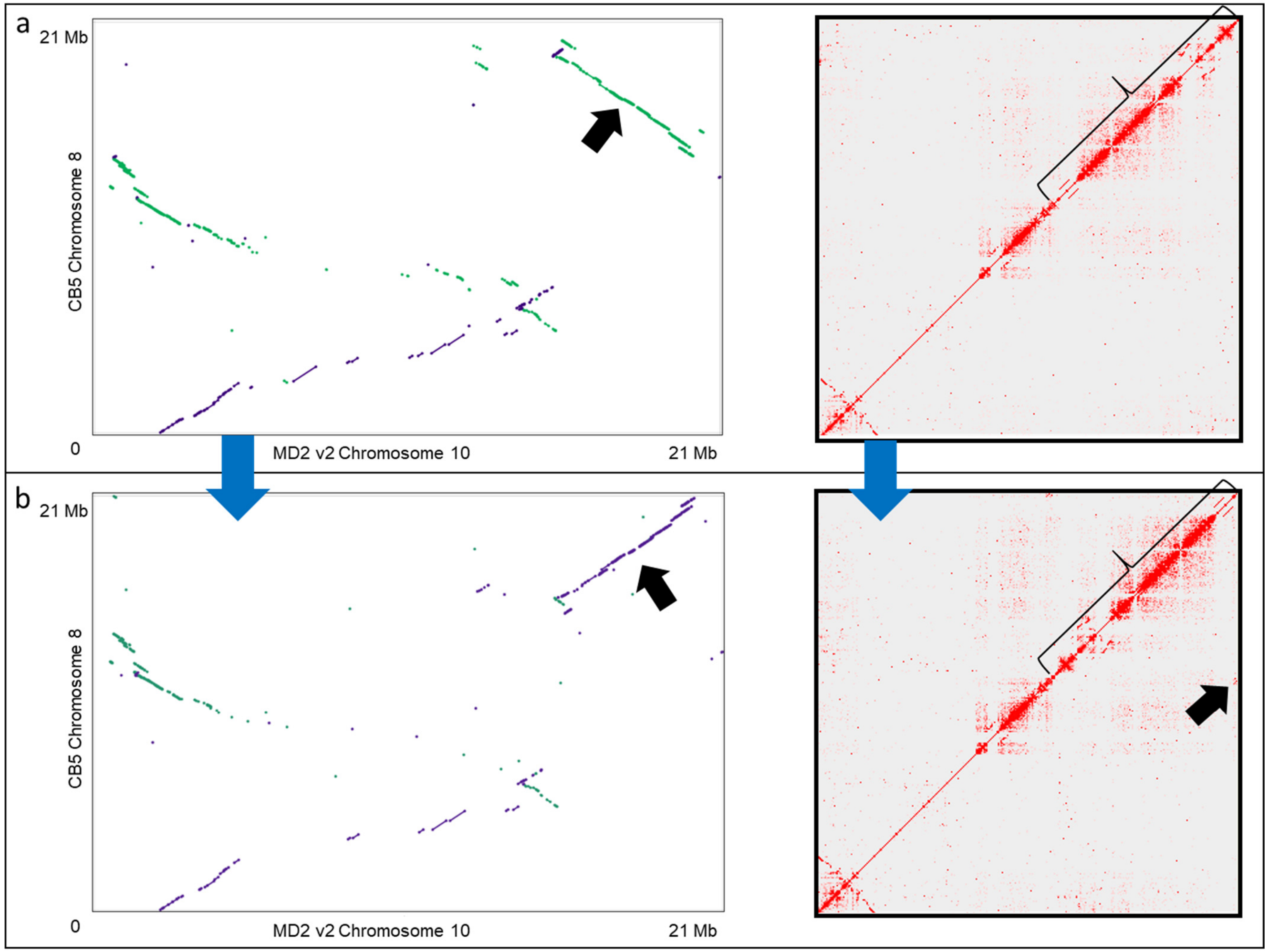

Figure S7. Manual inversions of non-collinear regions in CB5 chromosome 8. The MD2 v2 and CB5 genomes were aligned using Nucmer (left) and the MD2 Hi-C data was mapped to the CB5 genome (right). A non-collinear region of CB5 chromosome 8 (a) was manually inverted to establish a putative collinear region (b). After the manual inversion of the non-collinear region, the inverted region had stronger Hi-C interaction signals outside of the diagonal (see back arrow), indicating that these regions were more proximal, as in the original CB5 assembly and suggesting that the rearrangements represent true interspecific chromosomal differences between CB5 and MD2 genomes.

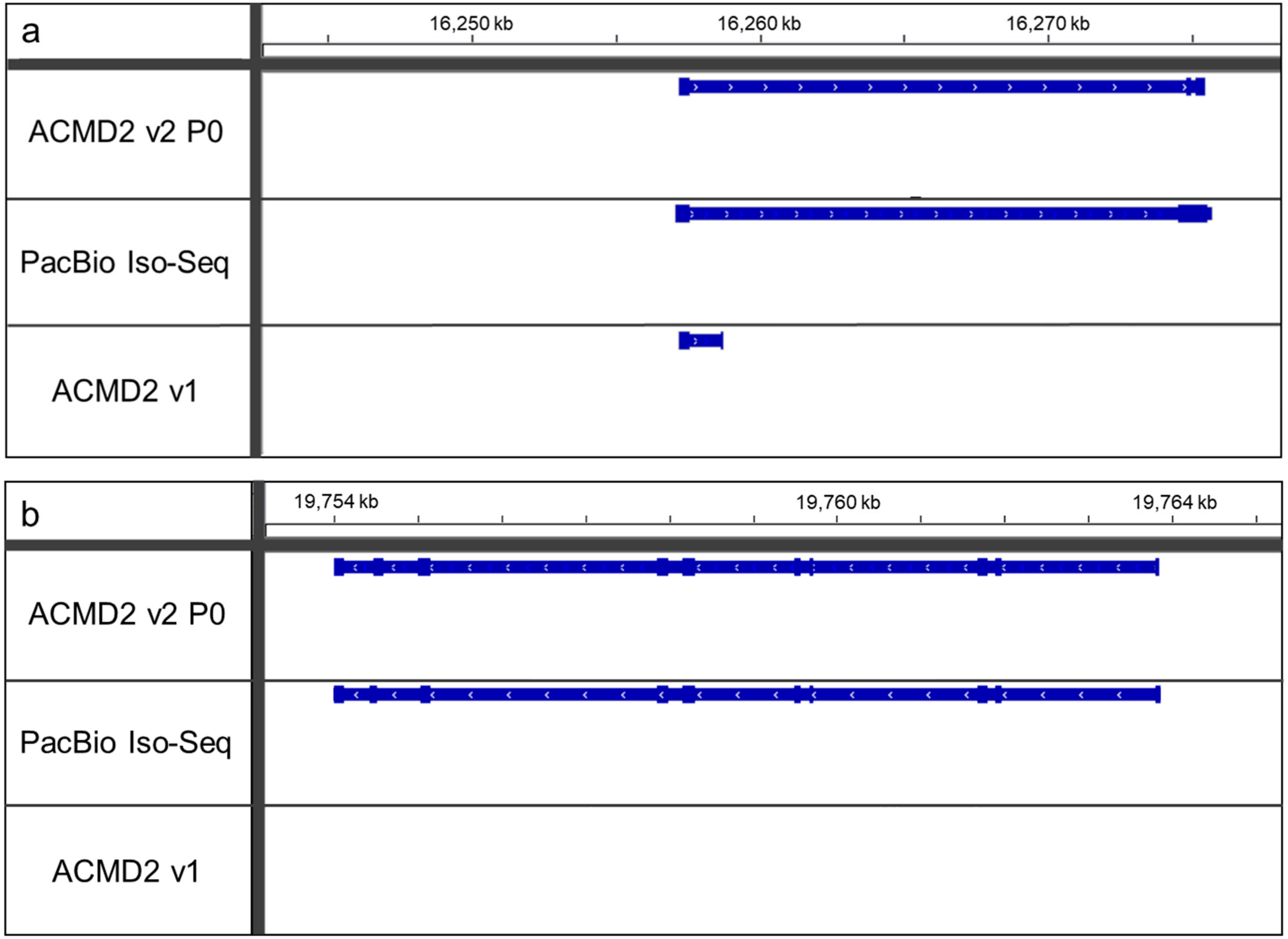

Figure S8. Evidence of Iso-Seq transcript data supporting the quality of the MDv2 gene prediction. This example also shows a gene that was predicted in the MD2 v2 assembly that had not been previously predicted in the v1 assembly. Panel (a) Demonstrates an example in which the Iso-Seq data supports the predicted gene structure in MD2 v2 compared to gene structure in v1 and (b) demonstrates an example in which the Iso-Seq data supports the prediction of a gene in MD2 v2 that was not previously predicted in the v1 assembly.

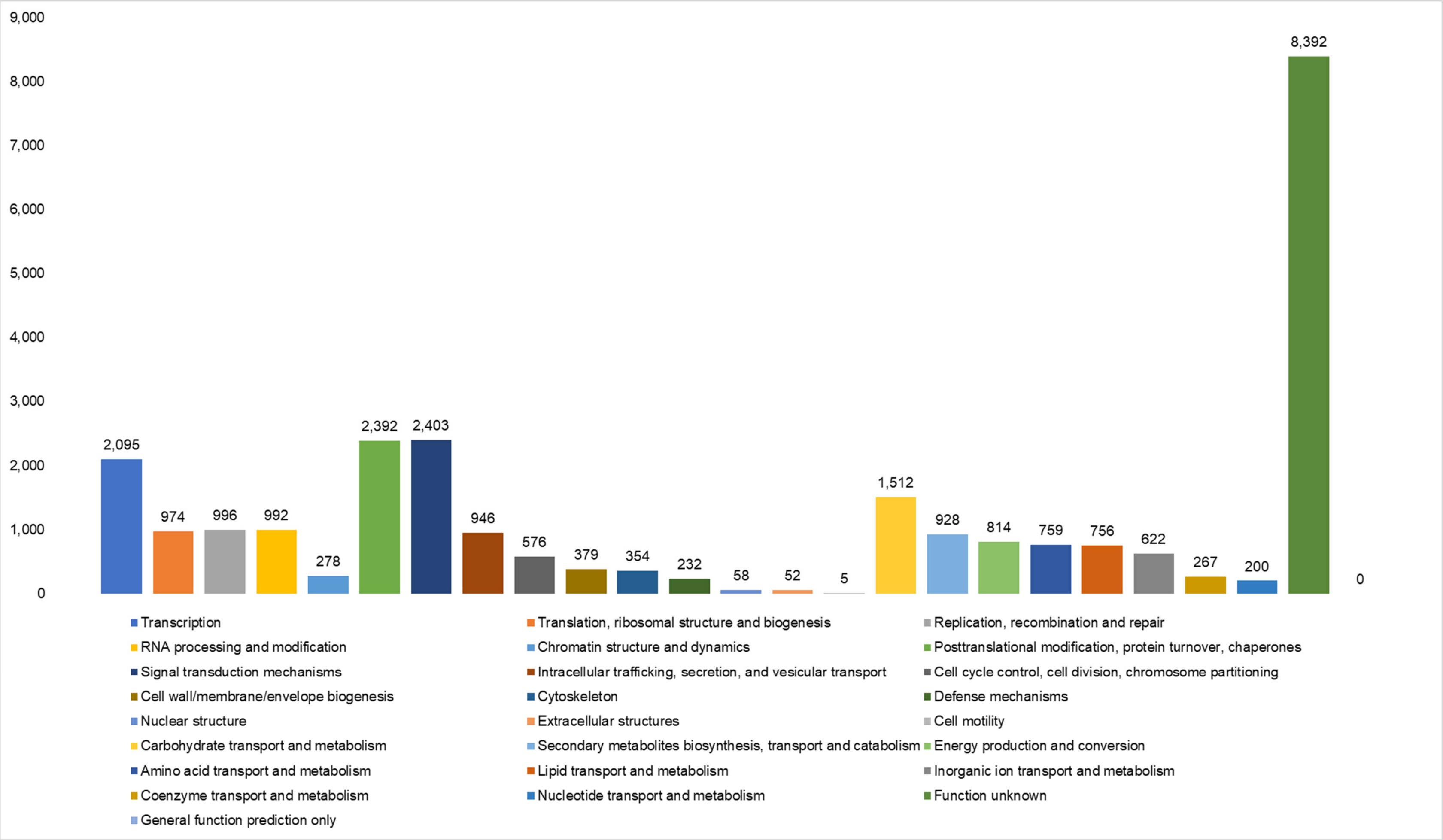

Figure S9. KOG (euKaryotic Clustering of Orthologous Groups) analysis of the complete set of genes for MD2 v2 P0. Query genes are placed into functional categories based on orthology to other functionally characterized genes in the KOG database.

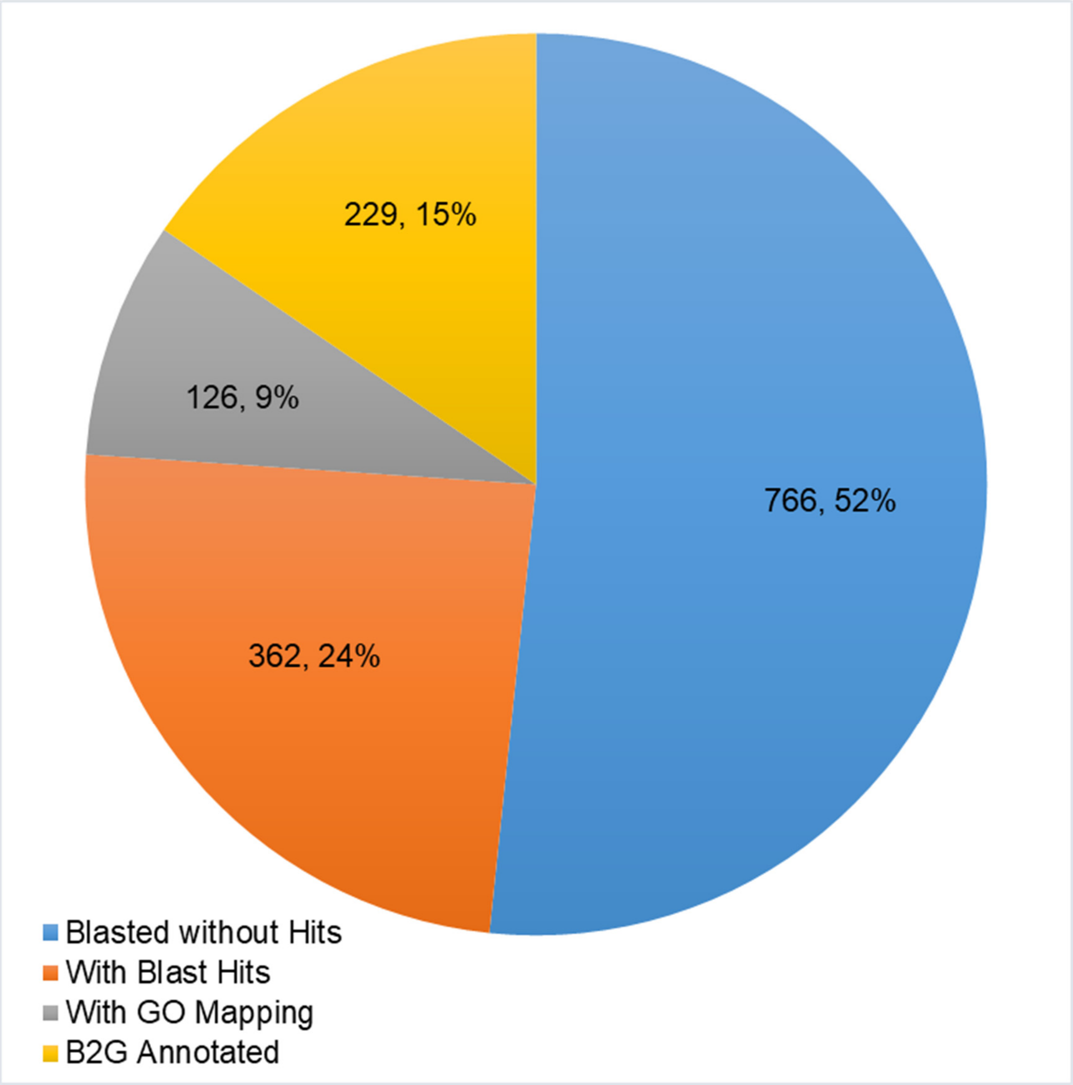

Figure S10. Data distribution for functional annotation results of novel pineapple genes with Blast2GO. A total of 1,483 new genes were identified in the MD2 v2 gene prediction that were not in previous pineapple (MD2 v1 and F153 v3) gene predictions.

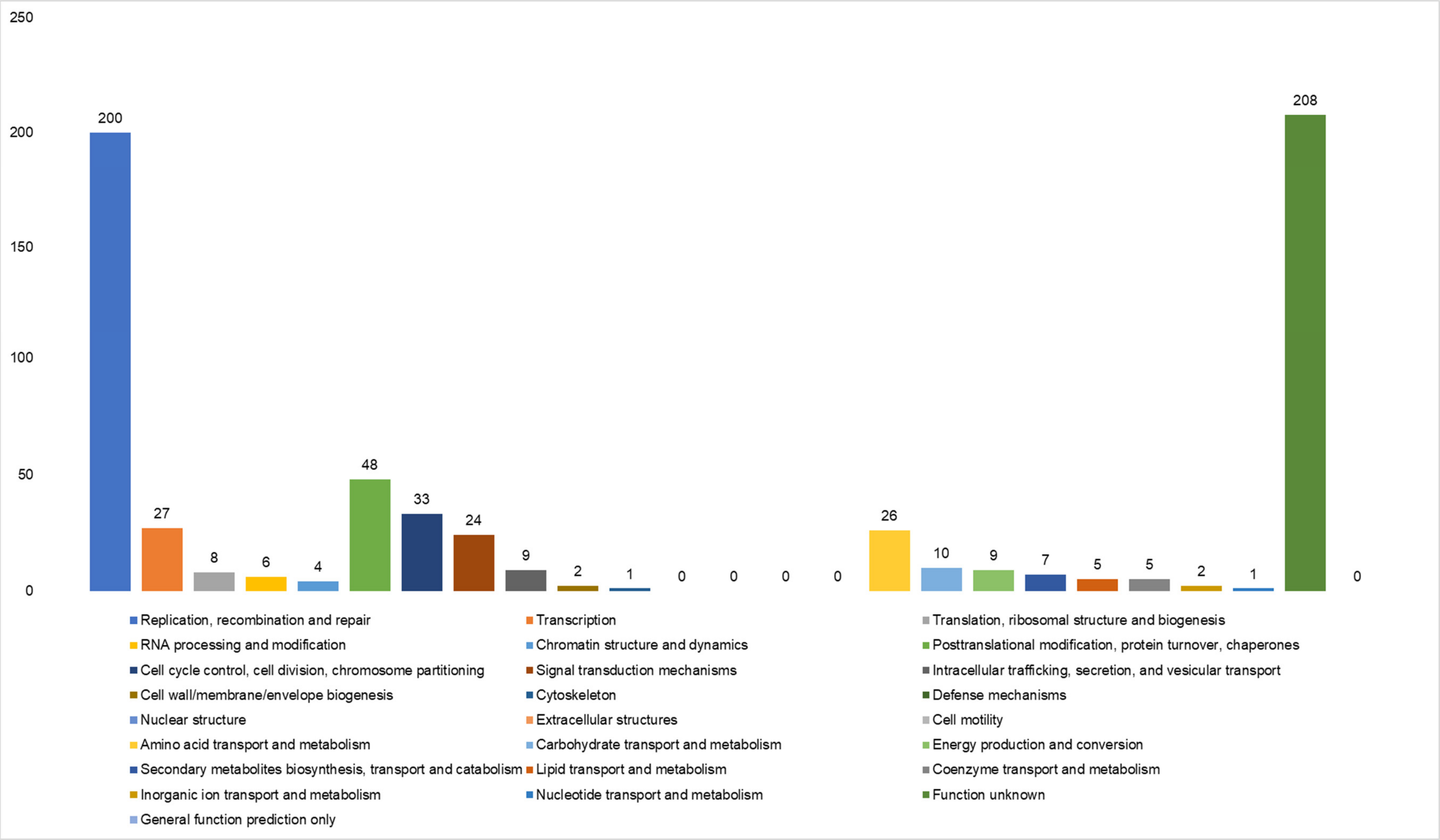

Figure S11. KOG (euKaryotic Clustering of Orthologous Groups) analysis of the novel subset of genes for MD2 v2 P0. These were genes identified in MD2 v2 that did not exist in previous pineapple (MD2 v1 and F153 v3) gene predictions. Query genes are placed into functional categories based on orthology to other functionally characterized genes in the KOG database.

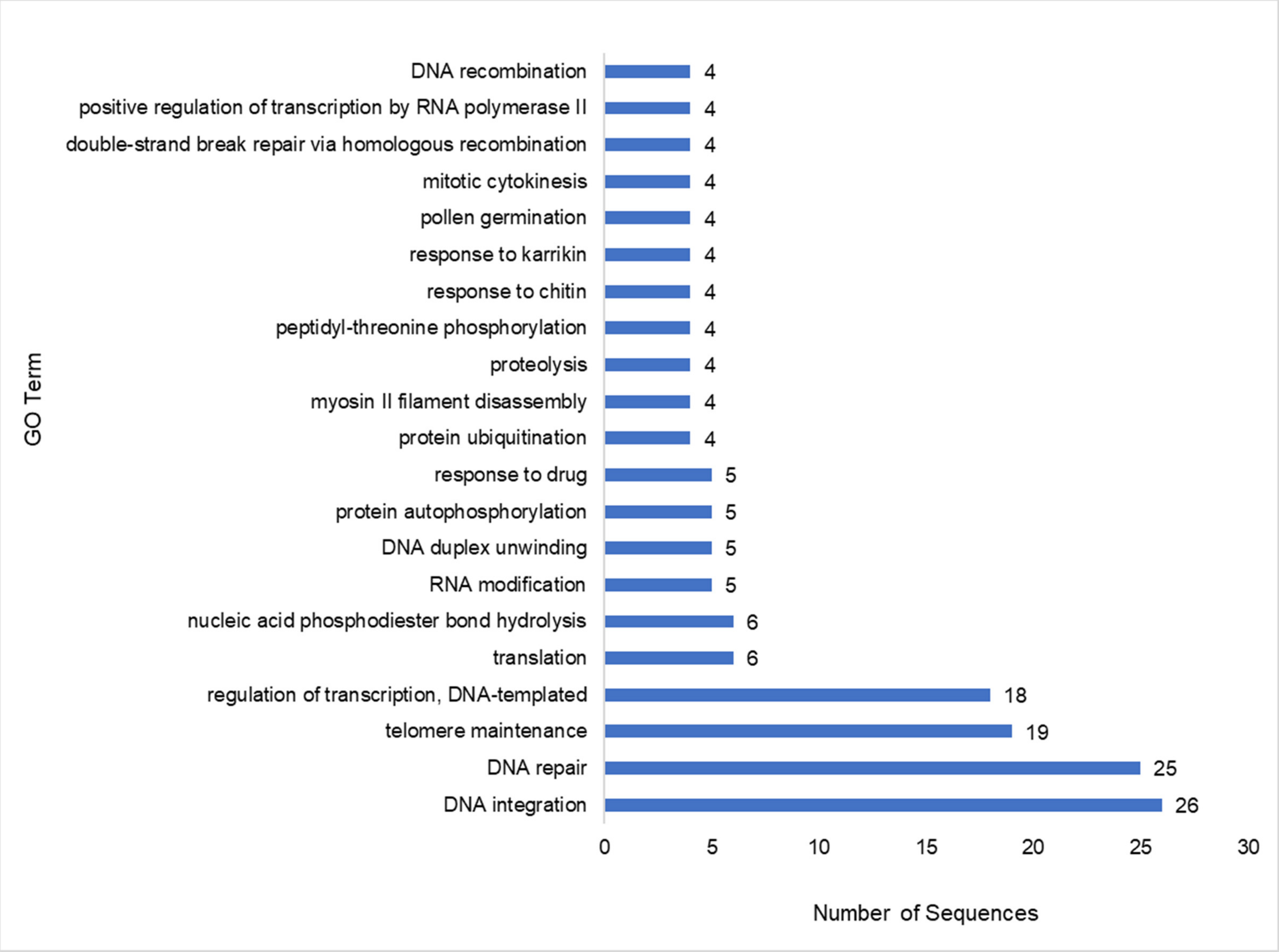

Figure S12. Direct biological process GO term counts for the novel genes identified in MD2 v2. A total of 1,483 genes were included in the GO term count, but only GO terms with >3 sequences are pictured.

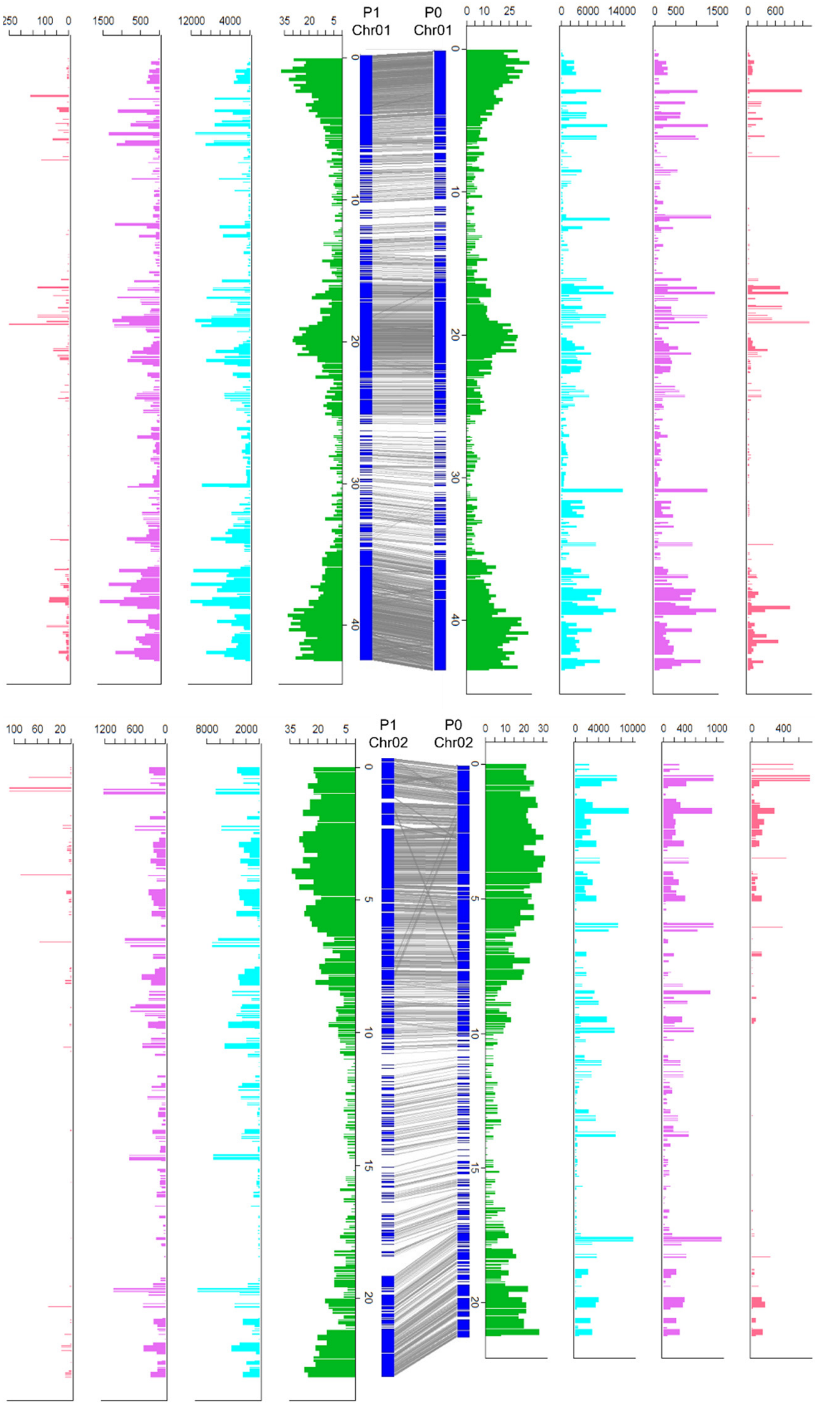

Figure S13. Haplotype diversity within the pineapple MD2 v2 genome. The central blue bars represent the two haplotypes of each chromosome in the pineapple genome. The gray lines indicate collinear genes. Outer plots include: distribution of genes (green), SNP density (cyan), INDEL density (pink), and density of potential deleterious effect variants (red). All numbers were determined in 200 kb windows.

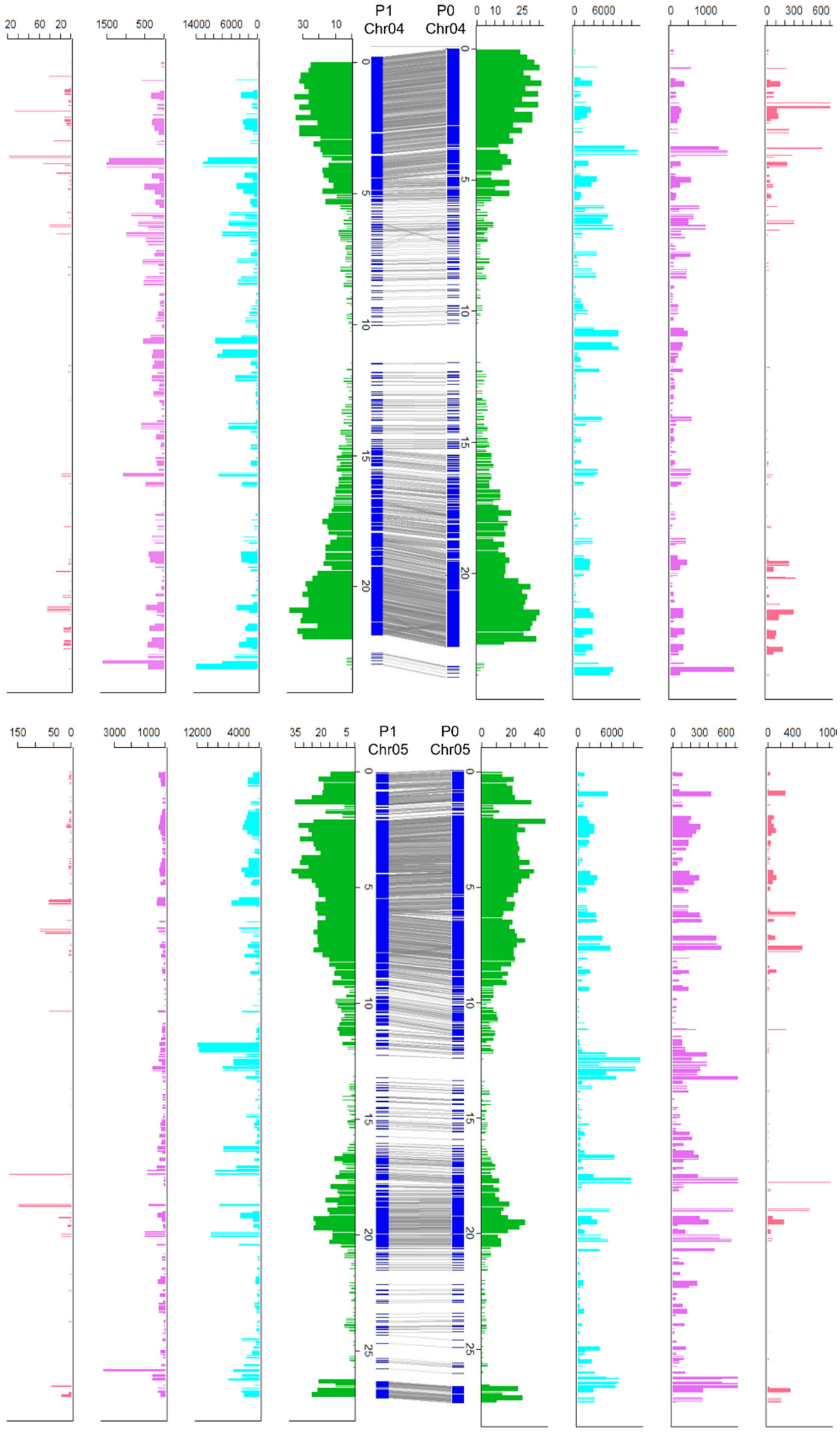

Figure S13 ***continued*.**

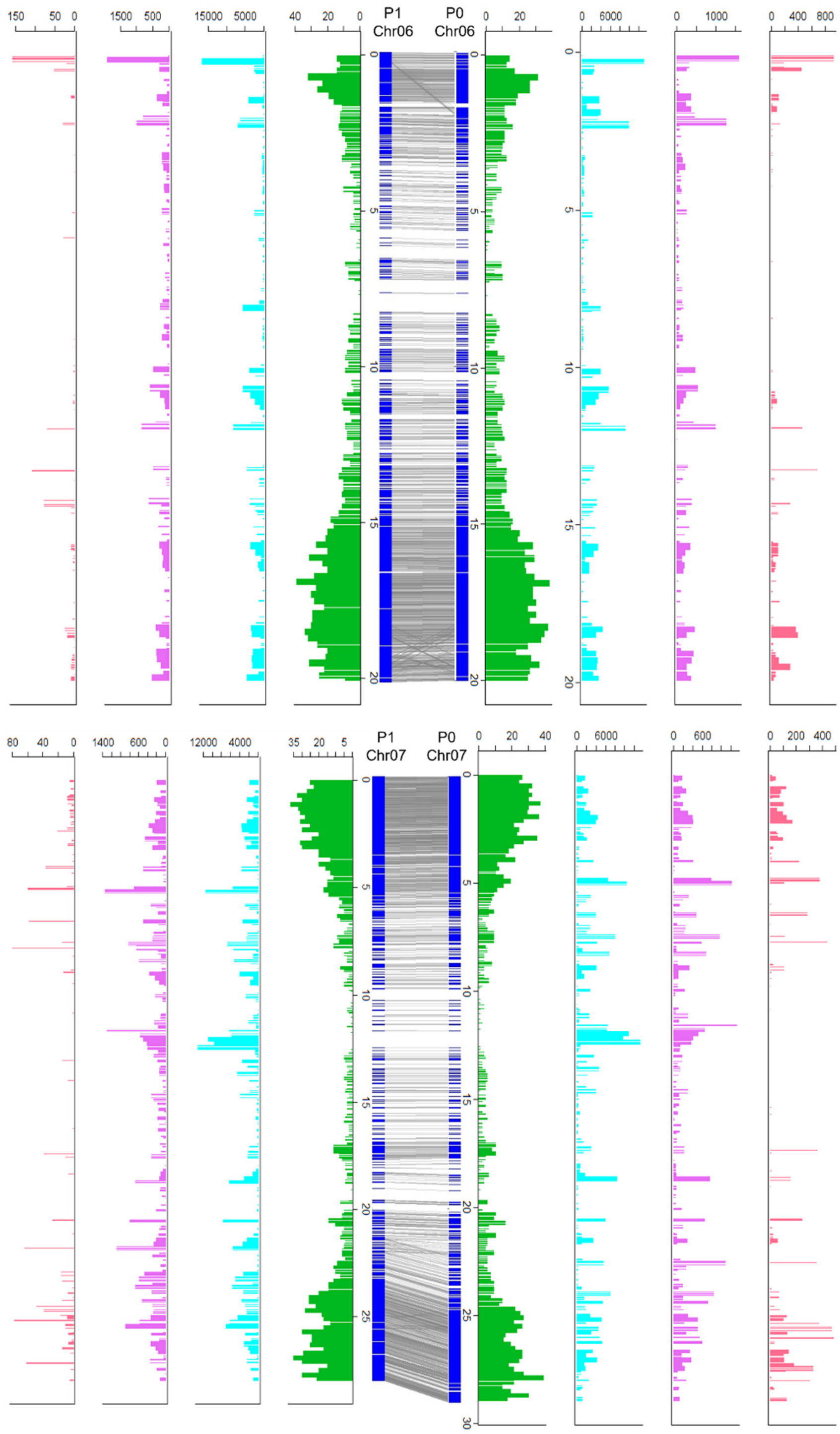

Figure S13 ***continued*****.**

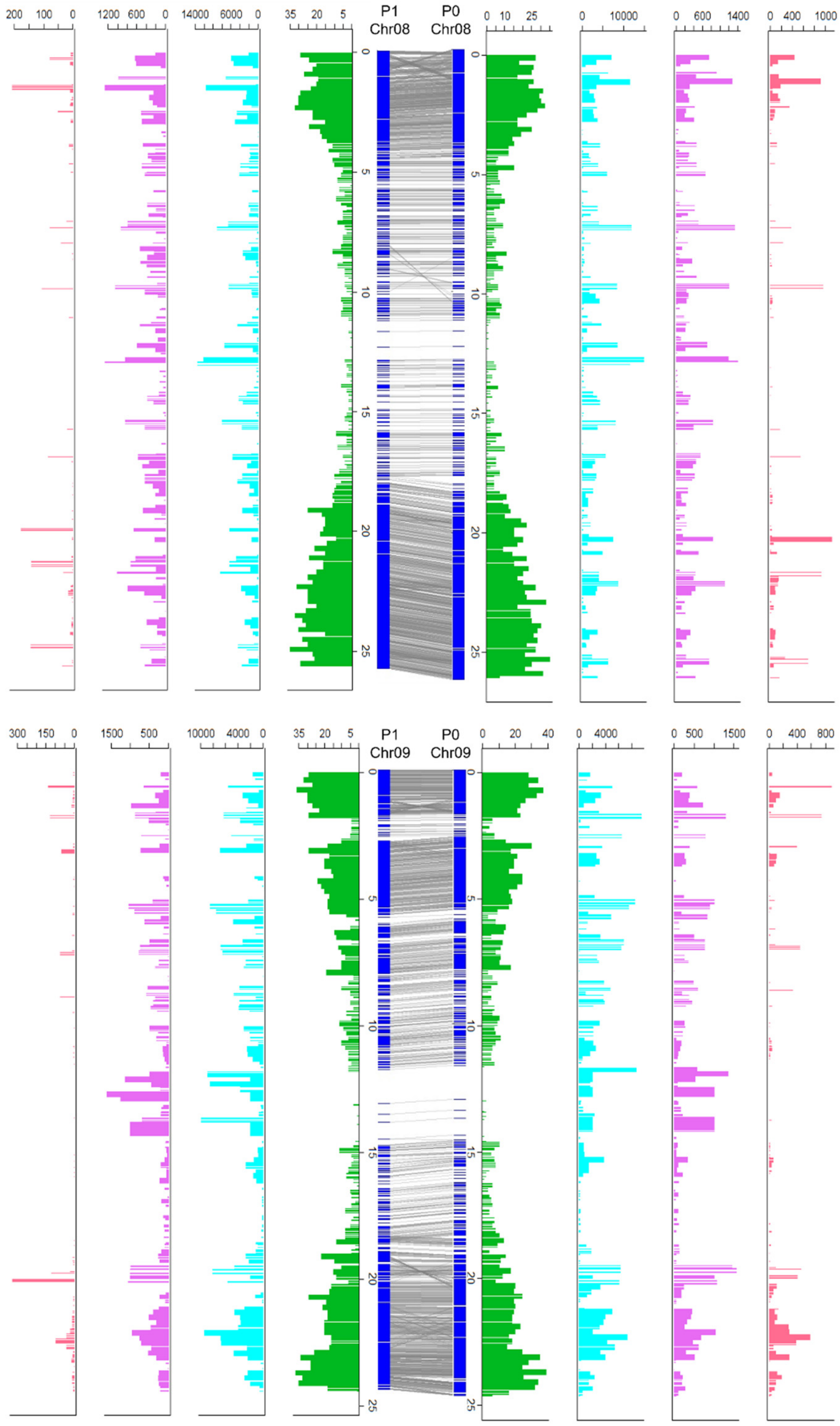

Figure S13 ***continued.***

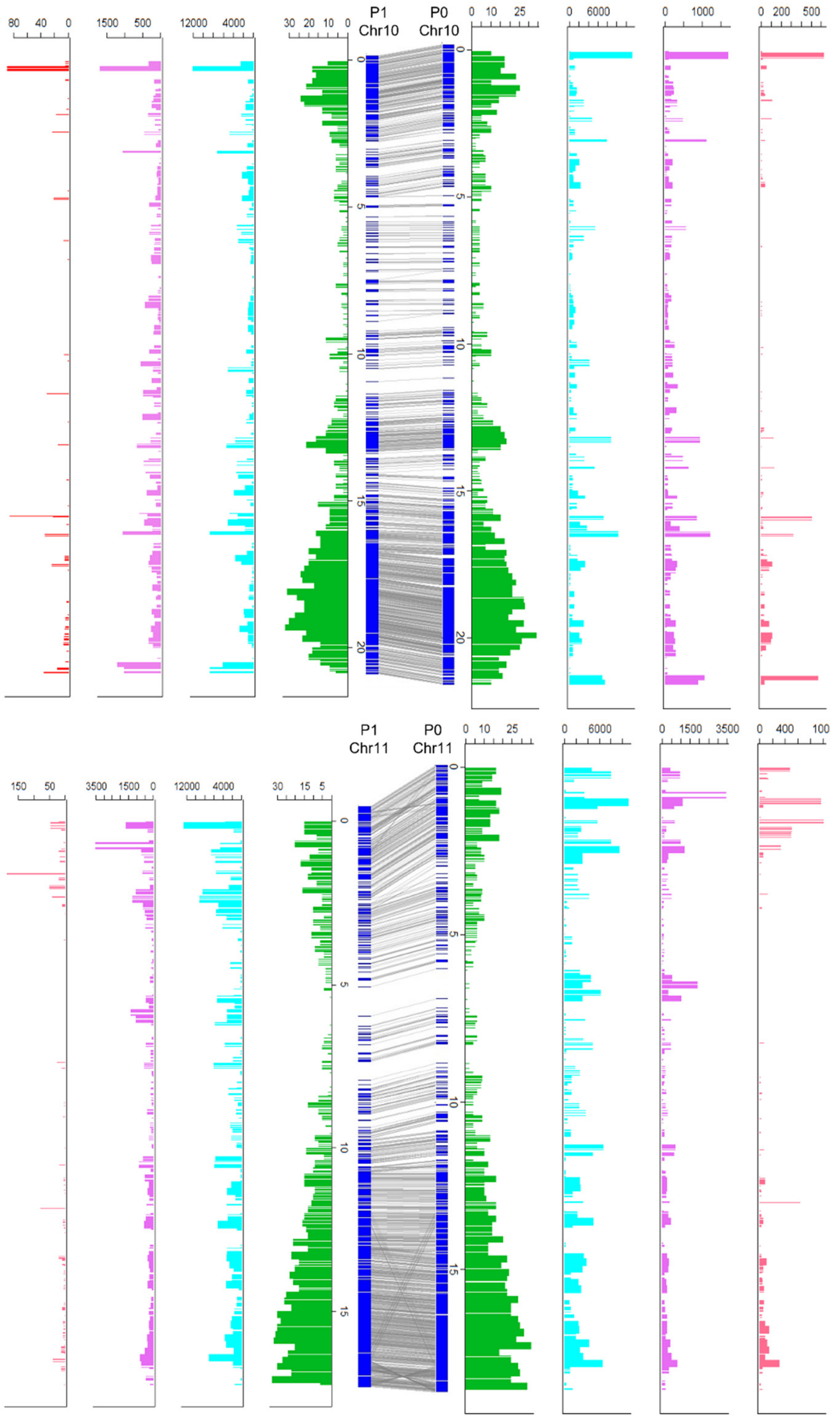

Figure S13 ***continued*.**

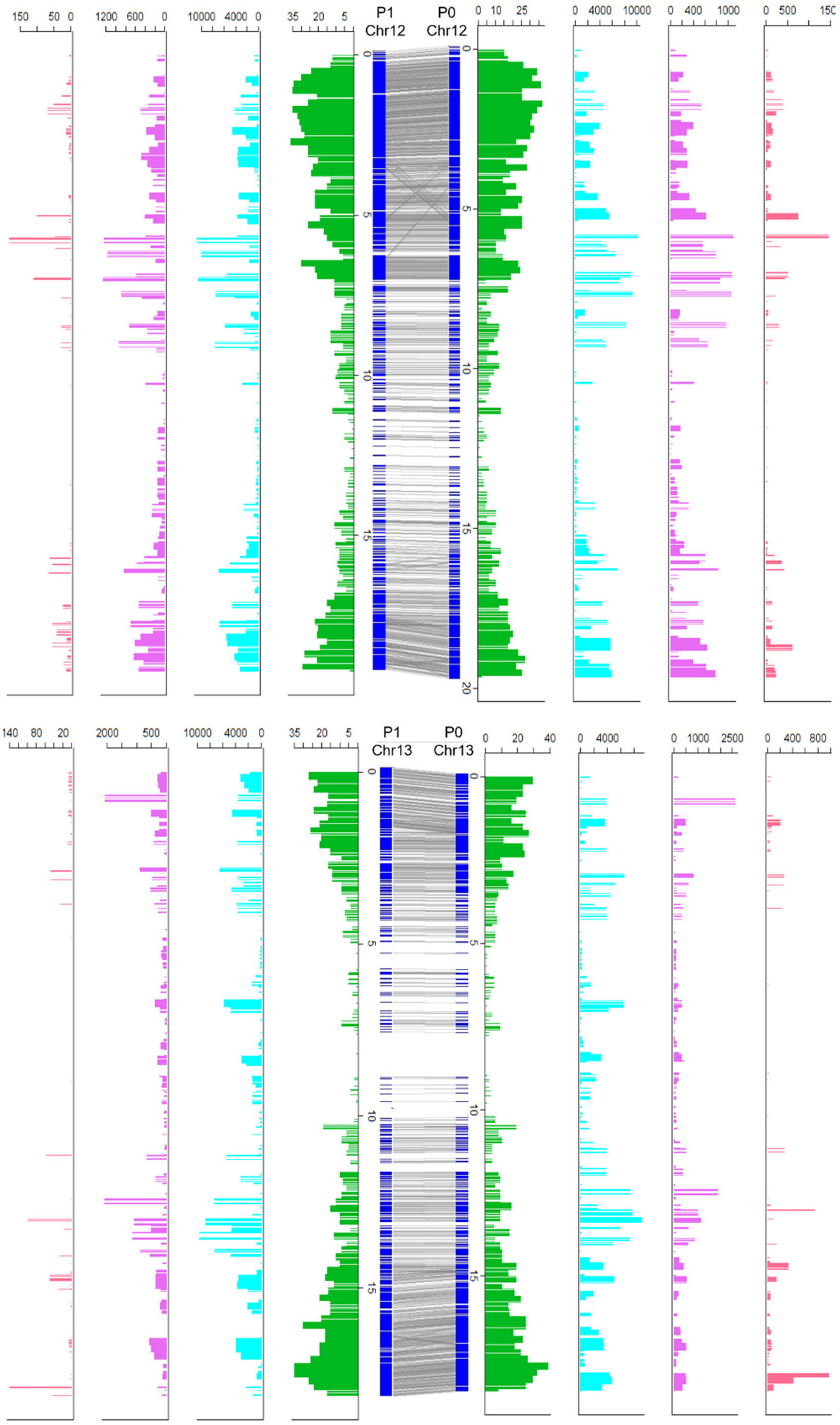

Figure S13 ***continued*****.**

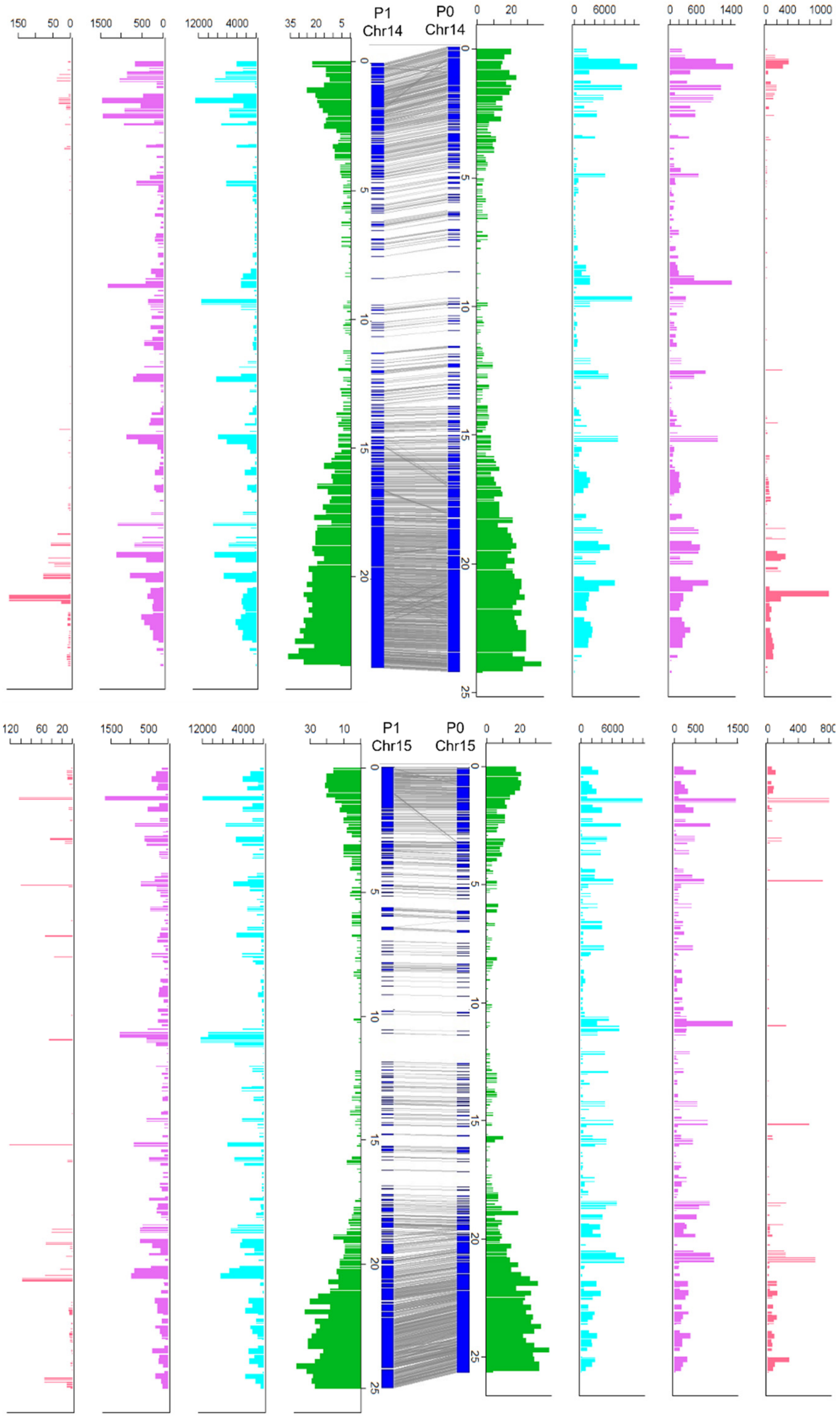

Figure S13 ***continued*****.**

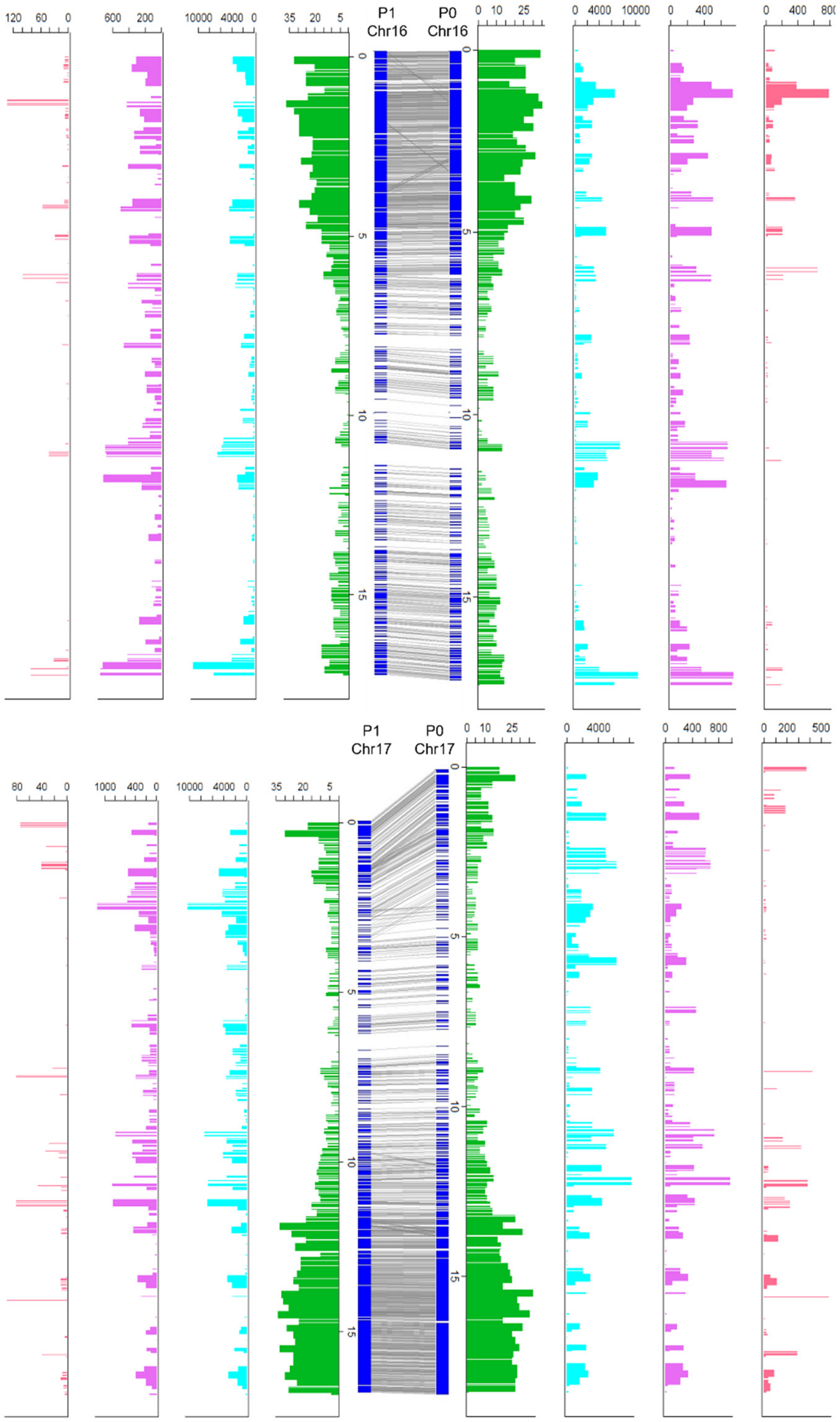

Figure S13 ***continued*****.**

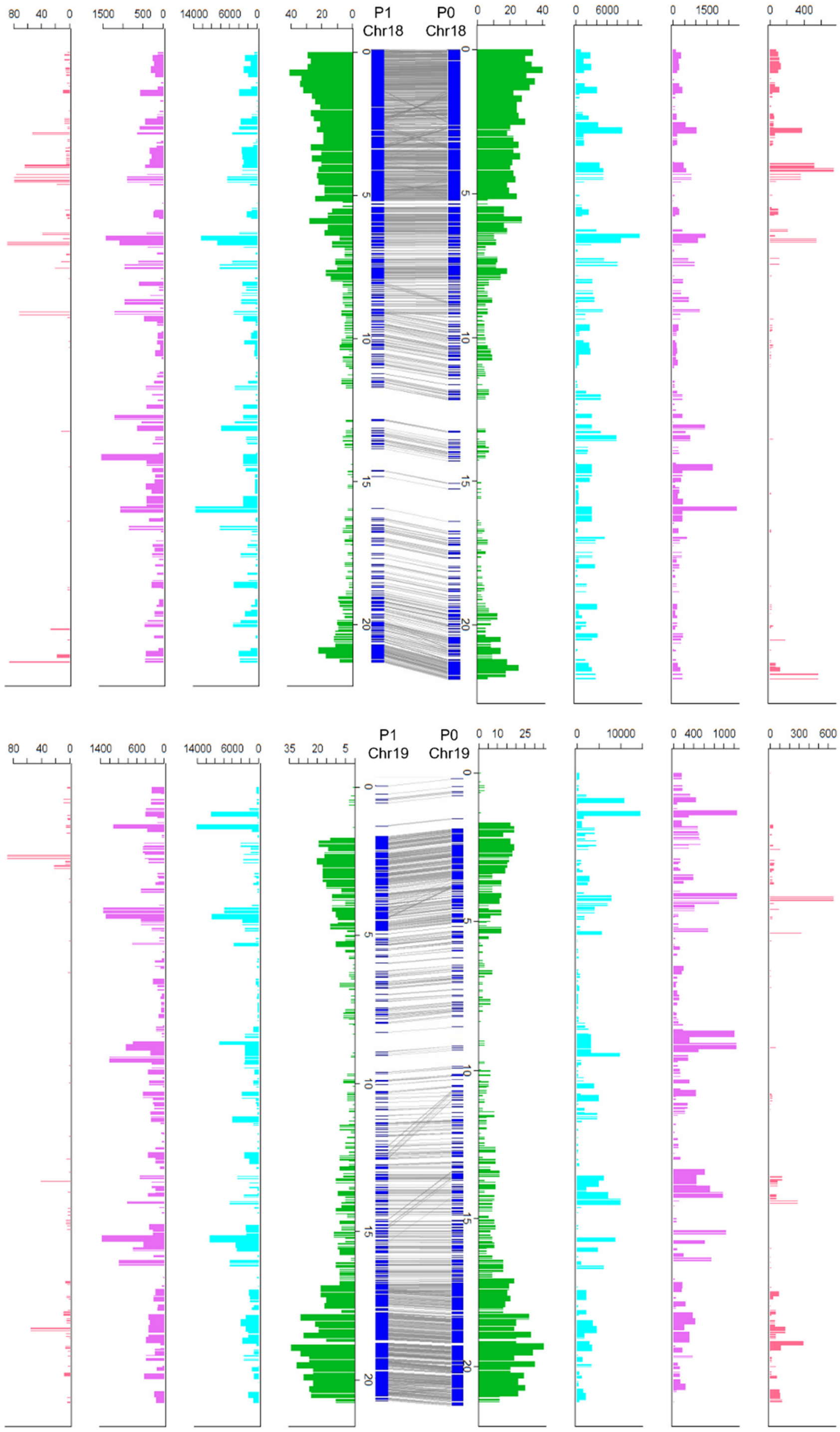

Figure S13 ***continued*****.**

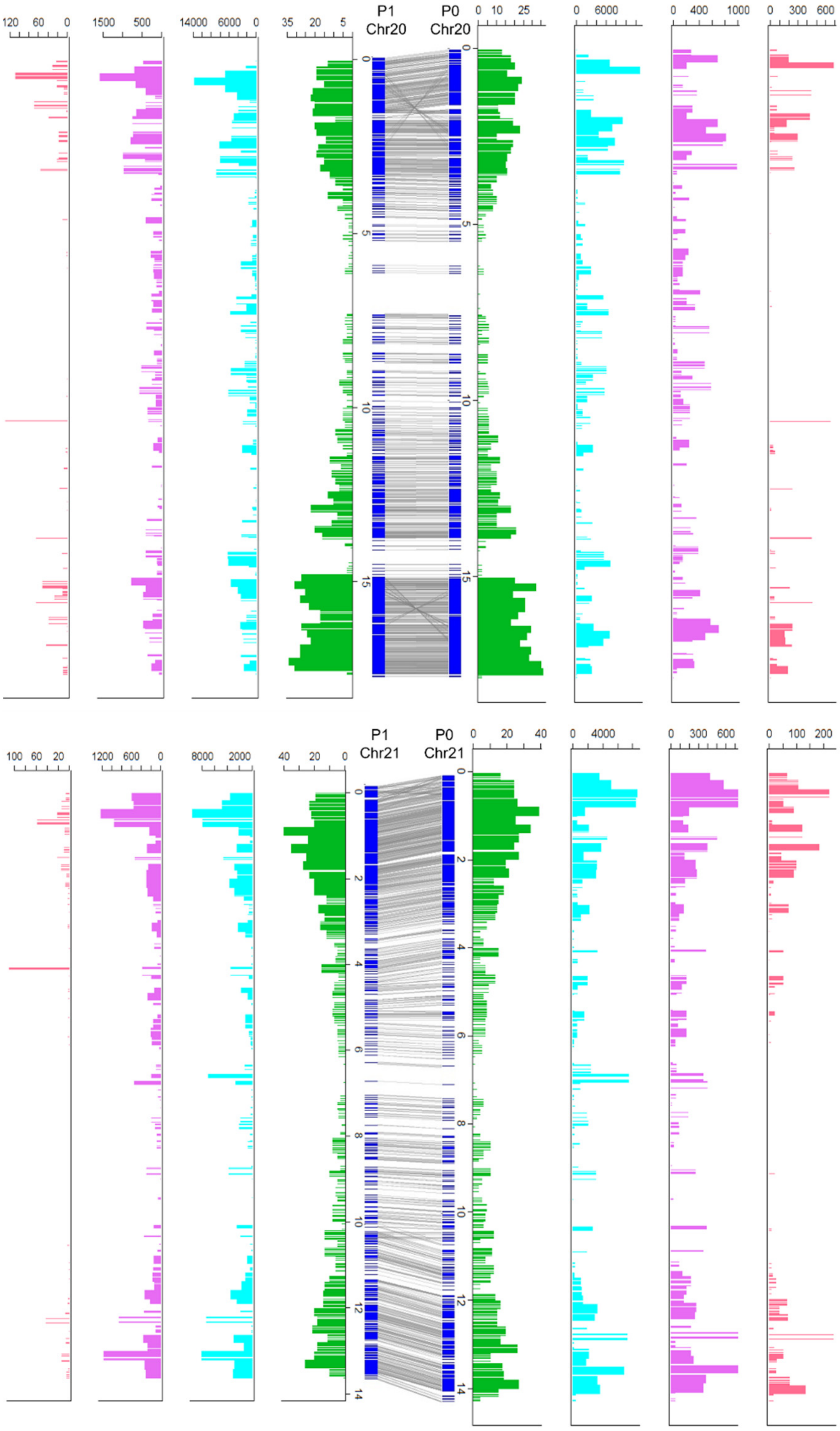

Figure S13 ***continued*.**

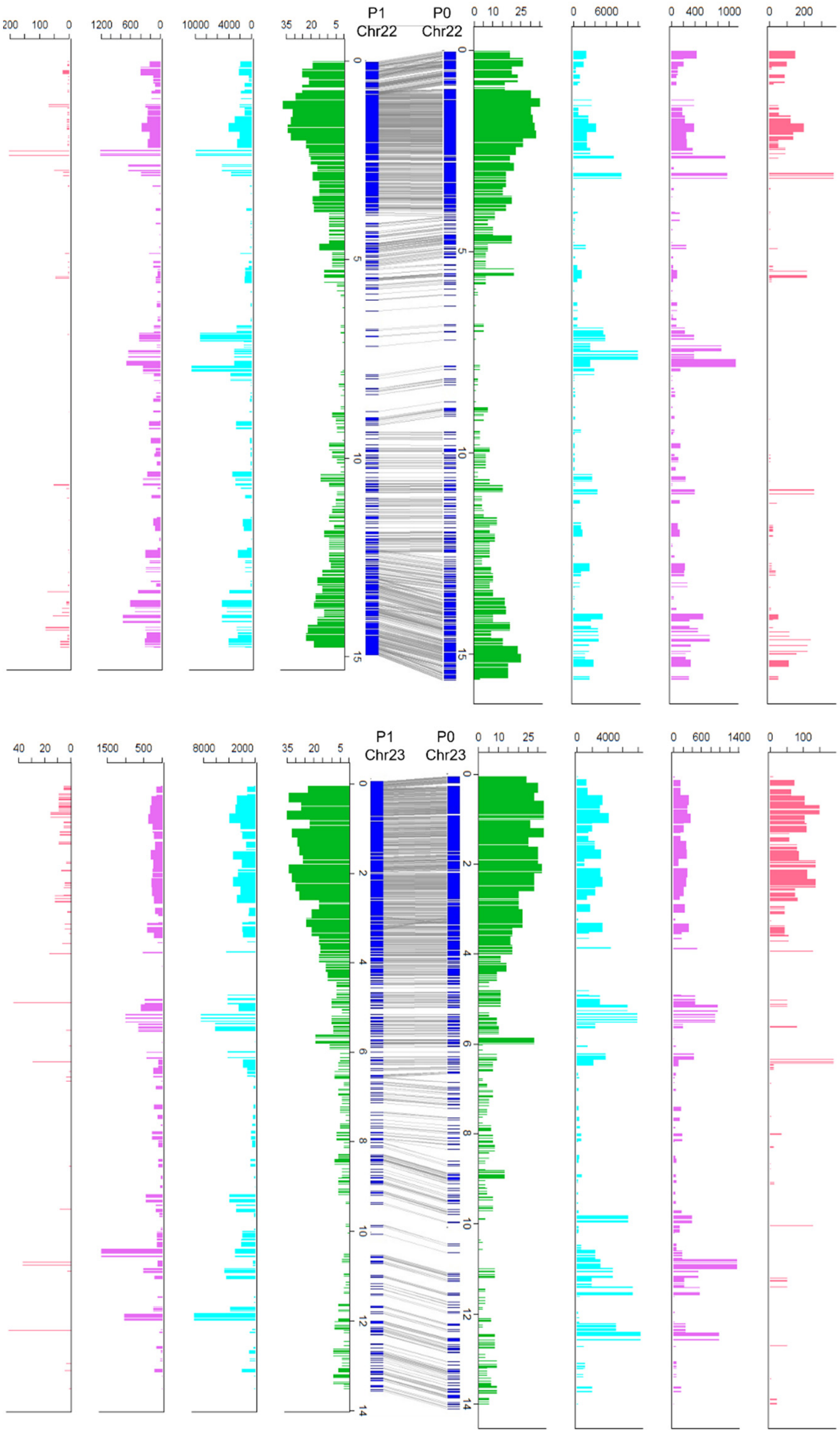

Figure S13 ***continued*.**

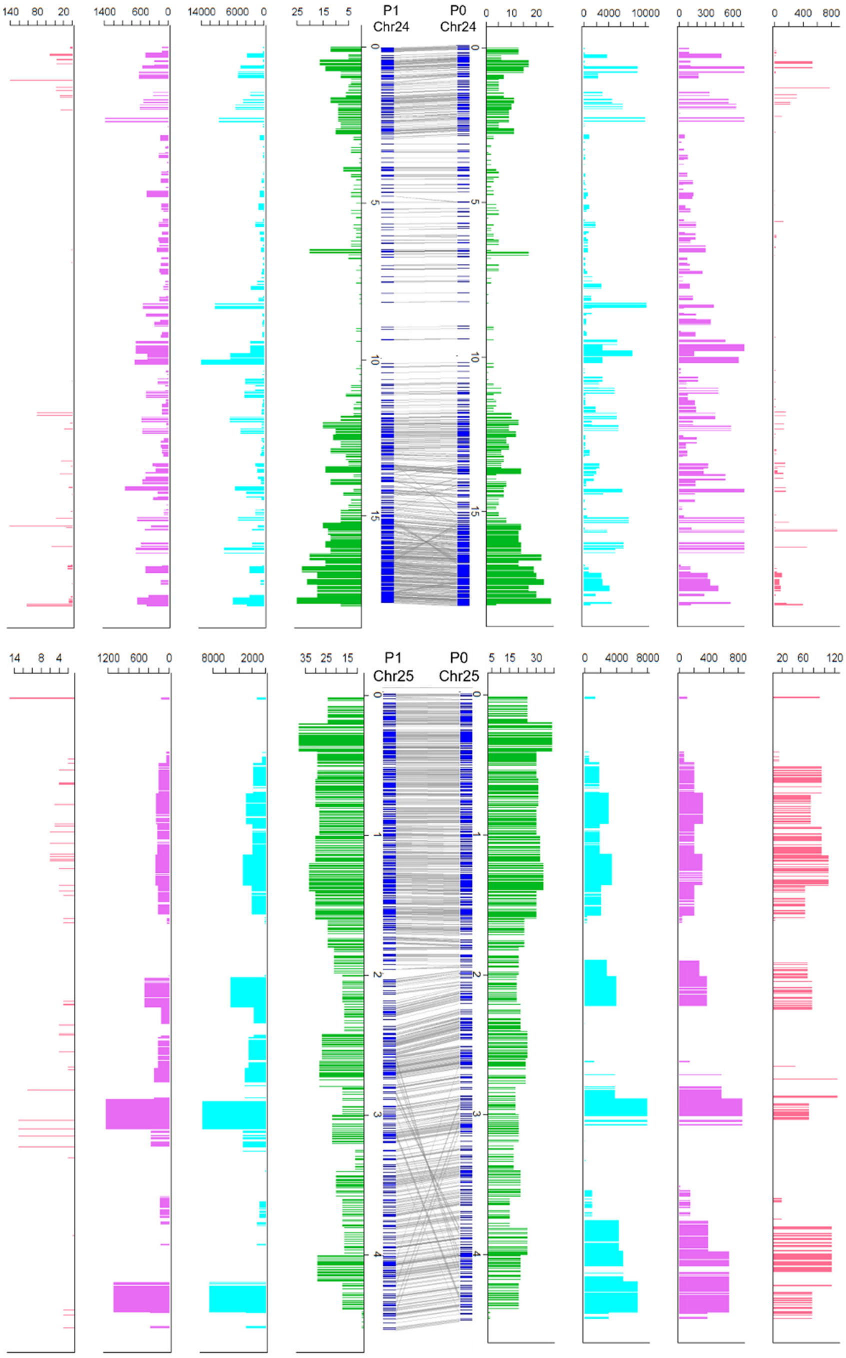

Figure S13 ***continued*.**

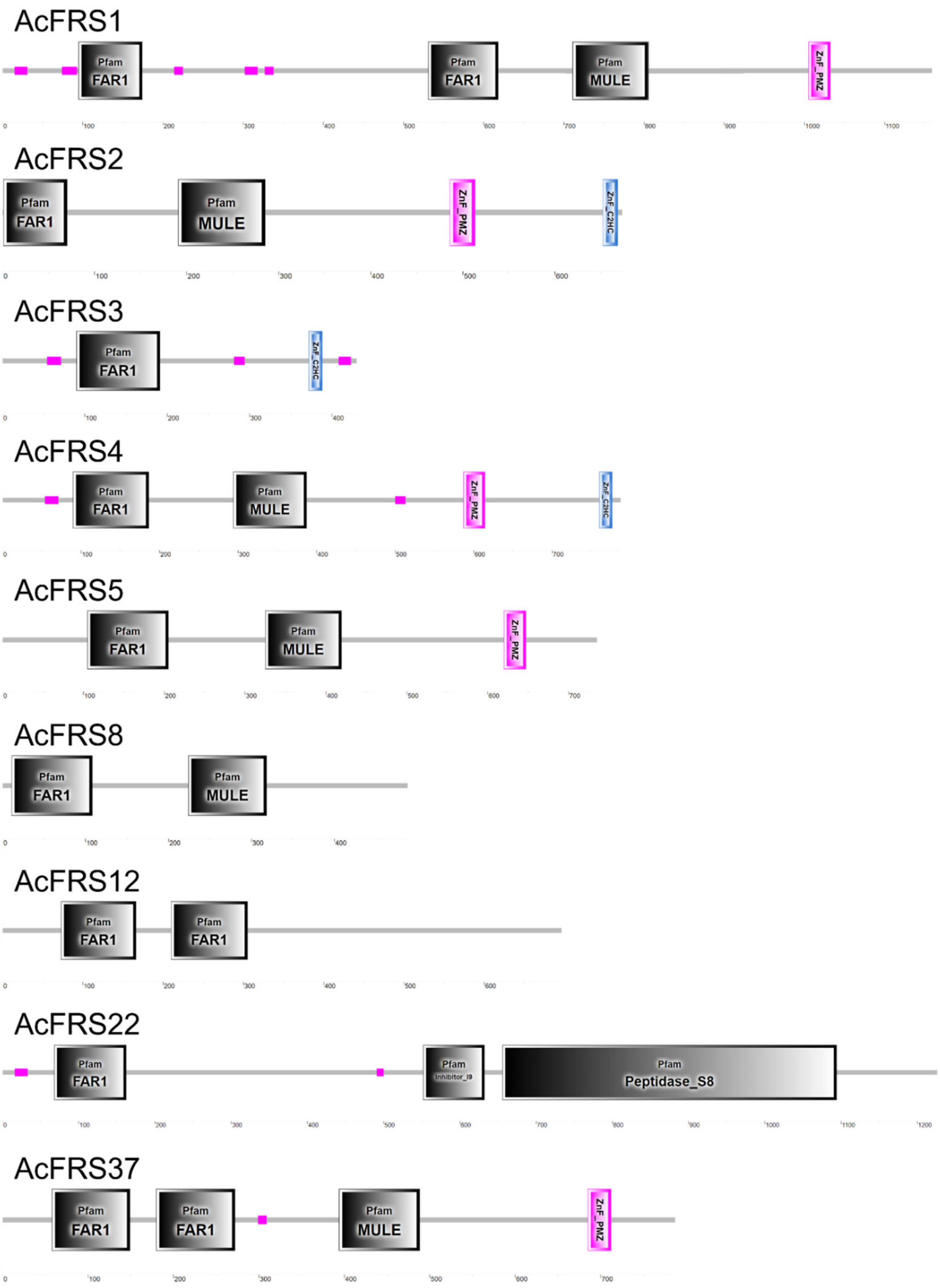

Figure S14. Representative protein structures for pineapple MD2 v2 FRS/FRF family genes (AcFRSs). AcFRS proteins all contain at least one FAR1 DNA binding domain (IPR004330). Other domains detected include the MULE transposase (IPR018289), Zinc finger, CCHC-type (IPR001878), and Zinc finger, PMZ-type (IPR006564) domains. Also of note, AcFRS22 contained Inhibitor_I9 (IPR010259) and Peptidase S8 (IPR036852) domains.
